# Anti-SARS-CoV-2 Antibodies Within IVIg Preparations: Cross-Reactivities With Seasonal Coronaviruses, Natural Autoimmunity, and Therapeutic Implications

**DOI:** 10.3389/fimmu.2021.627285

**Published:** 2021-02-17

**Authors:** Marinos C. Dalakas, Kleopatra Bitzogli, Harry Alexopoulos

**Affiliations:** ^1^Department of Neurology, Thomas Jefferson University, Philadelphia, PA, United States; ^2^Neuroimmunology Unit, National and Kapodistrian University of Athens Medical School, Athens, Greece

**Keywords:** IVIg, antibodies to SARS-CoV-2, autoreactivity, cross-reactivity with COVID-19, COVID-19

## Abstract

**Introduction:** Cross-reactivity to SARS-CoV-2 antigenic peptides has been detected on T-cells from pre-pandemic donors due to recognition of conserved protein fragments within members of the coronavirus's family. Further, preexisting antibodies recognizing SARS-CoV-2 with conserved epitopes in the spike region have been now seen in uninfected individuals. High-dose Intravenous Immunoglobulin (IVIg), derived from thousands of healthy donors, contains natural IgG antibodies against various antigens which can be detected both within the IVIg preparations and in the serum of IVIg-receiving patients. Whether IVIg preparations from pre-pandemic donors also contain antibodies against pre-pandemic coronaviruses or autoreactive antibodies that cross-react with SARS-CoV-2 antigenic epitopes, is unknown.

**Methods:** 13 samples from 5 commercial IVIg preparations from pre-pandemic donors (HyQvia (Baxalta Innovations GmbH); Privigen (CSL Behring); Intratect (Biotest AG); IgVena (Kedrion S.p.A); and Flebogamma (Grifols S.A.) were blindly screened using a semi-quantitative FDA-approved and validated enzyme-linked immunosorbent assay (ELISA) (Euroimmun, Lubeck, Germany).

**Results:** Nine of thirteen preparations (69.2%), all from two different manufactures, were antibody-positive based on the defined cut-off positivity (index of sample OD to calibrator OD > 1.1). From one manufacturer, 7/7 lots (100%) and from another 2/3 lots (67%), tested positive for cross-reacting antibodies. 7/9 of the positive preparations (77%) had titers as seen in asymptomatically infected individuals or recent COVID19-recovered patients, while 2/9 (23%) had higher titers, comparable to those seen in patients with active symptomatic COVID-19 infection (index > 2.2).

**Conclusion:** Pre-pandemic IVIg donors have either natural autoantibodies or pre-pandemic cross-reactive antibodies against antigenic protein fragments conserved among the “common cold” - related coronaviruses. The findings are important in: (a) assessing true anti-SARS-CoV-2-IgG seroprevalence avoiding false positivity in IVIg-receiving patients; (b) exploring potential protective benefits in patients with immune-mediated conditions and immunodeficiencies receiving acute or chronic maintenance IVIg therapy, and (c) validating data from a recent controlled study that showed significantly lower in-hospital mortality in the IVIg- treated group.

## Introduction

Efficient immune surveillance of infected or recovering populations from COVID-19 is important in the fight against the pandemic. Recent evidence suggests that anti-SARS-CoV-2 immunity not only occurs after an active infection, but may also precede an infection. Cross-reactivity to SARS-CoV-2 antigenic peptides has been detected on T-cells and B-cells from pre-pandemic donors owing to recognition of protein fragments conserved among common cold-related coronaviruses (1, 2). Spike protein-reactive CD4+ T cells are not only seen in 83% of patients with COVID-19 infection but also in 35% of healthy donors; further, spike-protein-reactive T cell lines from healthy donors respond similarly to the C-terminal region of the spike proteins of both, the human endemic coronaviruses as well as that of SARS-CoV-2 (2). Most importantly, antibodies cross-reacting to SARS-CoV-2 have not only been detected in healthy individuals (3) but, based on just published evidence, preexisting antibodies recognizing SARS-CoV-2 in uninfected individuals have conserved epitopes in the spike region targeted by neutralizing antibodies (4).

Intravenous Immunoglobulin (IVIg), derived from thousands of healthy donors, contains natural and cross-reactive IgG autoantibodies against various antigens at various thresholds of detection owing to accumulation of low antibody concentrations from single individuals. These antibodies can be detected at clinically significant levels not only within the IVIg preparations but also in the serum of IVIg-receiving patients (5). Whether IVIg preparations also contain antibodies that may cross-react with SARS-CoV-2 antigenic epitopes, is unknown. The information is important in accurately assessing anti-SARS-CoV-2-IgG seroprevalence, to avoid false positivity in IVIg-receiving patients, but also in exploring potential protective benefits in patients with immune-mediated conditions and immunodeficiencies receiving acute and chronic maintenance therapy (6).

## Methods

We examined if various commercial IVIg preparations contain anti-SARS-CoV-2-antibodies, due to cross-reactivity with pre-pandemic anti-coronavirus IgG antibodies or the existence of natural IgG antibodies, and accurately measured antibody titers to assess if they are clinically meaningful, compared to titers seen in COVID-19 infected patients.

We tested 13 samples from 5 commercial IVIg preparations using a semi-quantitative FDA-approved, enzyme-linked immunosorbent assay (ELISA) (Euroimmun, Lubeck, Germany) that measures anti-SARS-CoV-2-antibodies directed against the S1 viral spike protein. The assay, according to the manufacturer, has a 98.5 % sensitivity and 99 % specificity. An independent serological survey has validated this method reporting 93% sensitivity and 100% specificity (7). We have also used the same assay to screen serum and CSF from COVID-19 infected patients with encephalpathy (8) as well as COVID19 recovered patients and uninfected health-care workers (9). The cut-off positivity is set as the OD value measured at 450 nm divided by the OD value of the provided calibrator, being >1.1.

The following IVIg preparations were screened: HyQvia (Baxalta Innovations GmbH); Privigen (CSL Behring); Intratect (Biotest AG); IgVena (Kedrion S.p.A); and Flebogamma (Grifols S.A.). From one brand, 7 different lots were available and from another one 3 different lots. A total of 13 lots were screened. All preparations were coded by manufacturer and lot (A1-7, B1-3, C, D, E) and used as liquid preparations in either 1:250 or 1:500 dilution to match normal human serum IgG concentration (0.5 mg/ml). No dissolution was needed ensuring the lack or aggregate formation. No colloids were added as all IVIg preparations tested contained protective colloids.

Control sera were used from patients with various autoimmune neurological diseases, autoimmune neuropathies and non-COVID19 ICU-hospitalized patients from the serum Biobank of the Department of Pathophysiology, University of Athens Medical School. Ethical approval for Bio-banking had been granted from the University of Athens Ethics Committee.

## Results

Nine of thirteen preparations (69.2%), all from 2 different manufactures, tested positive based on the manufacturer's cut-off (index of sample OD to calibrator OD > 1.1) (Table 1). From one manufacturer, all 7 lots (100%) were positive (A1–A7-Table 1); from another, 2/3 (67%) lots (B1, B2) were positive while the third lot (B3-Table 1) was borderline (index 1.08) and we called it negative. Among the positive preparations, 7/9 (77%) had titers as seen in asymptomatically infected individuals or recent COVID19-recovered patients (8, 9) while 2/9 (23%) had higher titers with index > 2 (2.32 and 2.21 respectively), comparable to those seen in patients with active symptomatic COVID-19 infection as concurrently measured in the sera of 50 COVID-19 PCR-confirmed symptomatic patients (index range 1.19–8.47) (8, 9).

**Table 1:** IVIg preparations and antibody titers.

**IVIg preparations**	**Concentration of IgG**	**Anti-SARS-CoV-2 titers**
A1	100 mg/ml	1.46 - POSITIVE
A2	100 mg/ml	1.28- POSITIVE
A3	100 mg/ml	1.41- POSITIVE
A4	100 mg/ml	1.27- POSITIVE
A5	100 mg/ml	1.40- POSITIVE
A6	100 mg/ml	2.21- POSITIVE
A7	100 mg/ml	2.32- POSITIVE
B1	50mg/ml	1.38- POSITIVE
B2	50mg/ml	1.18- POSITIVE
B3	50mg/ml	1.08 - NEGATIVE
C	100mg/ml	0.89 - NEGATIVE
D	50mg/ml	0.66 - NEGATIVE
E	50mg/ml	0.74 - NEGATIVE

To further validate our results, we tested as controls pre-pandemic archived sera from: (a) 49 neurological patients with autoimmune neuropathies; only one had a titer of 2.32, comparable to COVID-19 infected patients and the IVIg-positive preparations (whether this patient had been previously receiving immunotherapy with IVIg is however unknown); and (b) 30 ICU-hospitalized patients; all were anti-SARS-CoV-2 antibody negative.

## Discussion

IVIg preparations, made from pre-pandemic healthy donors, exhibit cross reactivities with SARS-CoV-2 S1 antigens, with some preparations having clinically significant antibody titers comparable to those seen in COVID-19 infected patients. The findings suggest that the respective healthy IVIg donors had either natural autoantibodies or cross-reactive antibodies against antigenic protein fragments conserved among the other “common cold” -related coronaviruses, similar to the cross-reactivity observed for T-cells and B cells from unexposed donors (1, 2).

Being cognizant about false positivity, we ensured the reliability of the anti-SARS-CoV-2- antibody ELISA method by: (a) using an FDA-approved assay, same as in other major serological surveys (7–9); (b) testing pre-COVID-19 sera from patients with autoimmune diseases which are often polyreactive; and (c) testing 50 PCR-confirmed COVID-19 infected patients.

IVIg has been effectively used in some COVID-19 patients including COVID-19- immune inflammatory syndrome resembling Kawasaki's disease (10) but whether the antibodies detected in the present study have any protective or therapeutic effects in COVID-19-exposed populations is uncertain. A recent randomized placebo-controlled double-blind clinical trial with IVIg in severe COVID-19-infected patients showed that the in-hospital mortality rate was significantly lower in the IVIg group compared to the control group (11). Considering that acquired immunity may be short-lived in persons with mild or asymptomatic COVID-19 infection (12), the IVIg administrations currently prescribed as monthly maintenance for patients with immunodeficiency and immune-mediated diseases (6), may theoretically provide some added benefit, even though these natural or cross-reactive antibodies are not affinity maturated for SARS-CoV-2. Of interest, some of our elderly IVIg-receiving patients with autoimmune neuropathies have been reporting in follow-up visits that did well when infected by COVID-19, but we have no objective data to support any IVIg-derived benefit.

The just published evidence that preexisting seasonal coronavirus antibodies in uninfected individuals cross-react with SARS-CoV-2 recognizing conserved epitopes in the spike region targeted by neutralizing antibodies, may have important ramification for natural infection (4) and the potential role of IVIg. Because prior immunity induced by seasonal coronavirus can reduce the transmission of homologous and heterologous strains (4), enhancing the preexisting seasonal coronavirus-elicited immunity with the antibodies within the IVIg preparations may have an impact on the natural course of SARS-CoV-2 infection. Such a potential effect of IVIg may be also enhanced by the known additional multilevel actions of IVIg in autoimmunity, including cytokine neutralization, immunoregulatory T cells, co-stimulatory molecules, MHC-expression, complement and natural autoantibodies (6, 13).

Apart from a potential benefit, the observations are also important in assessing SARS-CoV-2 seroprevalence, emphasizing the need to ensure that sera from prior IVIg-treated patients may contain SARS-CoV-2-antibodies derived from IVIg rather than form acquired antiviral humoral immunity.

It would be of interest in a future study to check for antibodies against the S2 subunit when kits with verified reliability become available. Most importantly, it will be relevant to examine if the noted anti-SARS-CoV-2 antibodies have a neutralizing activity.

## Data Availability Statement

The original contributions presented in the study are included in the article/supplementary material, further inquiries can be directed to the corresponding author/s.

## Author Contributions

All authors contributed to conception and execution of the work, writing, and reviewing the manuscript.

## Conflict of Interest

The authors declare that the research was conducted in the absence of any commercial or financial relationships that could be construed as a potential conflict of interest.
